# Comparison of laryngeal mask airway supreme^TM^ as non-inflatable cuff device and self-pressurized air-Q^TM^ in children: Randomized controlled non-inferiority study

**DOI:** 10.1097/MD.0000000000014746

**Published:** 2019-03-08

**Authors:** Jagyung Hwang, Boohwi Hong, Yoon-Hee Kim, Won Hyung Lee, Yumin Jo, SooKyoung Youn, Chae Seong Lim

**Affiliations:** Department of Anesthesiology and Pain Medicine, College of Medicine, Chungnam National University, Daejeon, Korea.

**Keywords:** airway management, anesthesia, children, pediatrics, supraglottic device

## Abstract

**Background:**

: Supraglottic airway (SGA) device with non-inflatable cuff reduce the airway complications associated with cuff hyperinflation. The aim of the study is to determine whether the default setting of Supreme is as effective as the non-inflatable cuff devices. The oropharyngeal leak pressure was measured and compared between the Supreme and Air-Q, a typical non-inflatable cuff device. We hypothesized that the default setting of Supreme is non-inferior to the the Air-Q self-pressurized (SP) in respect to the oropharyngeal leak pressure.

**Methods:**

: Eighty-four patients aged 1 to 7 years who were scheduled for general anesthesia, participated in the study. The patients were randomly assigned to Supreme group (n = 41) or Air-Q SP group (n = 43). We considered that the primary outcome, oropharyngeal leak pressure of Supreme group would be non-inferior to the Air-Q SP group, within 3 cmH_2_O. Other outcomes included tidal volume loss, difficulty of insertion, insertion time, and complications.

**Results:**

: The oropharyngeal leak pressure of the Supreme and Air-Q SP was 19.9 ± 4.1 cm H_2_O and 17.4 ± 2.9 cm H_2_O, respectively. The mean differences of 2 devices (Air-Q SP—Supreme) were −2.5 cm H_2_O, (95% confidence interval [−4.0 to −0.9], *P* = .002). The upper CI was smaller than the non-inferiorty margin (3 cm H_2_O). This result suggested that the default setting of Supreme was superior to the Air-Q SP with respect to the oropharyngeal leak pressure. However, there were no significant differences in tidal volume loss over time, ease of device insertion score, insertion time, and complications.

**Conclusions:**

: The Supreme can be used in the default setting in pediatric patients accordingly in terms of tolerable leak pressure and the stability for mechanical ventilation compared with Air-Q SP.

## 1 Introduction

The supraglottic airway (SGA) devices are commonly used for general anesthesia as an alternative to endotracheal intubation^[1]^ because such devices are less invasive with respect to cardiovascular and respiratory system.^[2,3]^ The rate of use of SGA is now also increasing in pediatric patients^[4]^ because the devices allow safe management of upper respiratory infection,^[5]^ provide convenience of insertion and removal of the airway, and carry a low risk of airway injury during both inductions of general anesthesia and surgery.^[6,7]^

Several SGA devices are available for use in children.^[8]^ These devices can be classified according to whether they have an inflatable cuff or non-inflatable cuff. Conventionally, SGA devices with inflatable cuff are used, however, the intracuff pressure has been an issue for pediatric clinicians because of the potential for airway morbidity due to excessive intracuff pressure.^[9,10]^ The fact that for various reasons most clinicians do not routinely measure the intracuff pressure further exacerbate the problem.^[10]^ In order to prevent such complications, the use of the SGAs with non-inflatable cuff is recommended because they may reduce airway morbidity associated with cuff hyperinflation and do not need intracuff pressure measurement.^[11–13]^

The Supreme is a single-use SGA consisting of an elliptical airway tube, inflatable cuff, and drainage tube.^[14]^ It has advantages of gastric access and tube fixation, whereby the mask can be fitted securely to the face.^[15]^ The cuff of the Supreme is maintained in slightly inflated state when assembled in the factory manufacturing settings. According to the manufacturer's instructions, the Supreme is to be inserted after deflation and provide additional inflation after insertion. Although the Supreme is an excellent device with many advantages, the handling of the inflatable cuff can create a problem when used in pediatric patients. The aim of the study is to determine whether the default setting of Supreme is as effective as the non-inflatable cuff devices. The oropharyngeal leak pressure was measured and compared between the Supreme and Air-Q, a typical non-inflatable cuff device. We hypothesized that the default setting of Supreme is non-inferior to the Air-Q self-pressurized (SP) in respect to the oropharyngeal leak pressure.

## 2 Materials and methods

Before performing the study, we received Institutional Review Board approval from the hospital's ethics committee and registered the trial at the Clinical Research Information Service. Written informed consent was obtained from the parents of all cases. This prospective, randomized, controlled trial was performed between May 2016 and January 2017 at Chungnam National University Hospital (Daejeon, Republic of Korea). Children, aged 1 to 7 years of American Society of Anesthesiologists physical status 1 or 2, were scheduled for elective general surgery and orthopedic surgery, which SGA devices would be suitable for airway management. The operation time was approximately 1 hour. Exclusion criteria included patients with active respiratory symptoms such as rhinorrhea, cough, and fever on the day of surgery; developmental delays; history of laryngopharyngeal diseases; gastroesophageal reflux disease; patients expected to have a difficult airway and full stomach; and patients who refused to participate in this study.

A total of 84 children were randomly assigned to the Supreme group or Air-Q SP group using a computer-generated random number table with blocks size of 2 and 4, according to a 1:1 ratio. For group allocation concealment, random number table was uploaded to redcap (secure web application for building and managing online databases; redcap.cnuh.co.kr), and group allocation was performed immediately before general anesthesia. Then the SGA devices were prepared. A researcher who did not participate in SGA insertion, anesthesia, or outcome evaluation performed the randomization. The patients, their parents, and the researcher performing the outcome evaluation were all blinded to the group assignment.

Before induction of anesthesia, all of the patients were pre-medicated by intravenous (iv) injection of glycopyrrolate 0.004 mg·kg^−1^. Standard monitors including electrocardiography, pulse oximetry, non-invasive blood pressure, and capnography were placed. After induction of general anesthesia with ketamine (1.5 mg·kg^−1^ iv) and rocuronium (0.6 mg·kg^−1^ iv), we inserted SGA devices. Each device was lubricated with a lidocaine-based gel before insertion. Two anesthesiologists performed insertion of both SGA devices. They had experienced in inserting SGA devices more than 100 pediatric patients.

The Supreme was inserted without deflation or additional air inflation, which differed from the manufacturer's instructions. The cuff of Supreme was maintained slightly inflated state of the factory manufacturing settings. We selected device sizes based on the patient's weight (Supreme: size 1.5, 5–10 kg; size 2, 10–20 kg; size 2.5, 20–30 kg. Air-Q SP: size 1.5, 7–17 kg; size 2.0, 17–30 kg; size 2.5, 30–50 kg). Successful device insertion was confirmed by movement of both chest walls, auscultation, and a stable capnography wave. The patient was maintained with 2% to 3% sevoflurane in 50% oxygen. Mechanical ventilation was provided with a tidal volume of 8 mg·kg^−1^, fresh gas flow of 3 l·min^−1^ respiratory rate of 14–20 breaths·min^−1^, and end-tidal carbon dioxide concentration of 30 to 40 mmHg. A neuromuscular blocking agent was not further administered. We also recorded hemodynamic changes such as heart rate, blood pressure, and saturation immediately before and after insertion, 1 minute and 5 minutes after insertion, and just before device removal.

Oropharyngeal leak pressure was measured as the primary outcome. Secondarily, tidal volume loss, insertion of device score, insertion time, and complications were compared. Oropharyngeal leak pressure was measured as the pressure when the expiratory valve closed with a fresh gas flow of 3 l min^−1^ until equilibrium was reached,^[16]^ or pressure when a leak sound was heard around the device and then was released. Tidal volume loss was measured based on the percentage of inspiratory volume (set)—expiratory volume (outcome), which was subsequently graded. We recorded the tidal volume loss just after insertion, 1 minute and 2 minutes after insertion, immediately after incision, and just before removal of the device. If there was no leakage, the sample was graded as 1, minor leakage (tidal volume loss <10%) was graded as 2, moderate leakage (10% <tidal volume loss <20%) was graded as 3, and insufficient sealing (tidal volume loss >20%) was graded as 4. In addition, the value was expressed as 0% when the percentage of tidal volume loss (%) was (−) value.

The ease of insertion was graded from 1 to 4 (no resistance at the time of insertion, mild resistance, insertion was successful on the second attempt, and insertion failed in the second attempt and endotracheal intubation was performed).^[17]^ Insertion time was checked from when the facemask was removed to the first capnography upstroke after insertion.^[18]^ Complications including cough, laryngospasm, desaturation (SpO_2_ <90%), gastric insufflation, and vomiting were recorded.

The required sample size was calculated based on a preliminary 10 patients who were inserted with the Air-Q SP. Leak pressure of these patients is 17 cmH_2_O with a standard deviation of 4. According to a similar study, the difference in leak pressure greater than 6 is significant and considered as clinically important.^[19]^ In this study, the difference in leak pressure of 3 was decided as the noninferiority margin, achieve a power of 90% with a risk of 0.025 for type 1 errors, and the minimum number of patients required in each group was 38. The total of 84 patients was included to allow for a dropout rate of 10%. One-sided non-inferior testing for primary outcomes was performed by comparing the 95% CI of the difference between groups for the leak pressure to the predetermined noninferiority margin (3 cm H_2_O).

All statistical analyses were performed using R statistical software (version 3.4.2: R Core Team, Vienna, Austria), and the normality of the data was assessed with the Shapiro-Wilk test. For comparison between groups, if the normality was satisfied, we used the independent *t* test and the results were expressed as the mean ± SD. When the normality was not satisfied, the variables were expressed as the median (quartile), and the Mann–Whitney *U* test was used to compare values of the 2 groups. Categorical data were compared using the chi-squared test. Repeated measurements (tidal volume loss %) were analyzed using repeated measures analysis of variance. A 2-tailed *P* value <.05 was considered statistically significant.

## 3 Results

**Figure 1:**
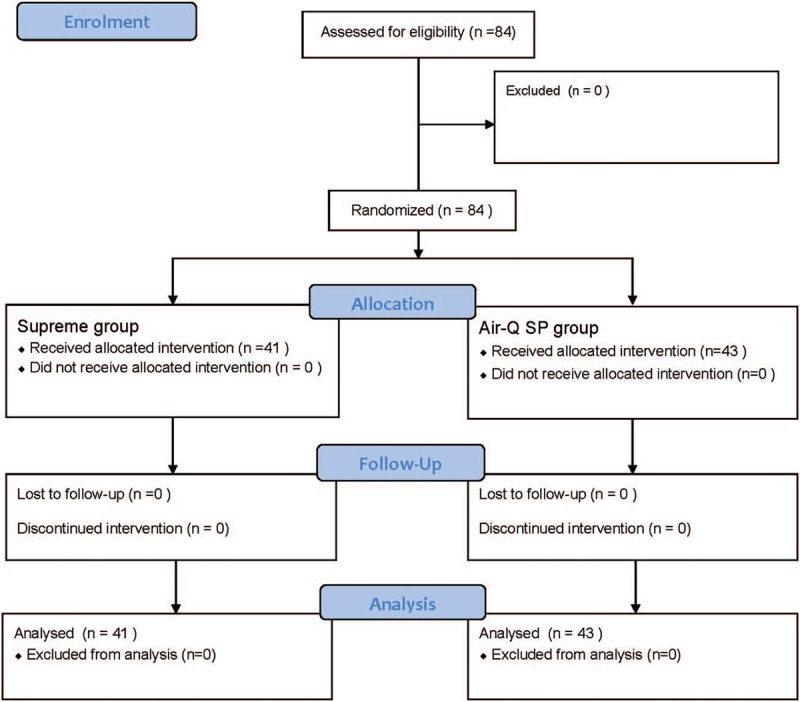
CONSORT figure representing enrollment data. CONSORT = Consolidated Standards of Reporting Trials.

A total of 84 patients consented and participated in the study. No patients declined to participate or were excluded after enrollment. A total of 41 and 43 patients were assigned to the Supreme group and Air-Q SP group, respectively, and all patients were analyzed (Fig. 1). Table 1 shows the patient characteristics of the 2 groups. The oropharyngeal leak pressure of the Supreme and Air-Q SP was 19.9 ± 4.1 cm H_2_O and 17.4 ± 2.9 cm H_2_O, respectively. The mean differences of 2 devices (Air-Q SP—Supreme) were −2.5 cm H_2_O, (95% confidence interval [−4.0 to −0.9], *P* = .002). The upper CI was smaller than the noninferiorty margin (3 cm H_2_O). This result suggested that the default setting of Supreme was superior to the Air-Q SP with respect to the oropharyngeal leak pressure.

**Table 1:**
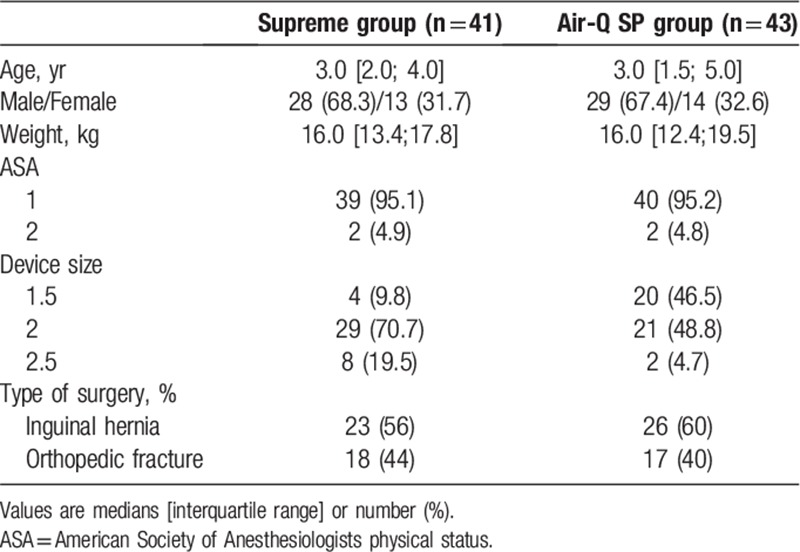
Patient characteristics and induction profiles.

**Figure 2:**
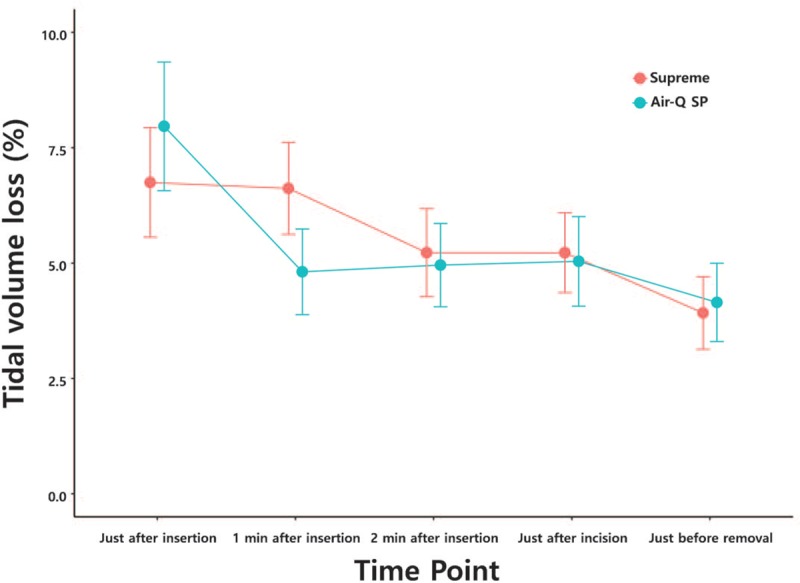
Changes in tidal volume loss (%) over time. There was no statistically significant difference in changes in tidal volume loss over time between the 2 groups. (group: time point *P* value = .135).

Tidal volume loss did not show a significant difference between the 2 groups at all measurement points (Table 3, Fig. 2). In addition, there were no significant differences with regard to ease of insertion (insertion of device score) and insertion time between the 2 groups (Table 2). There was more resistance, and a second attempt was required more often in the Air-Q SP group (3 cases) compared to the Supreme group (1 case), but the differences were not significant (Table 2). Hemodynamic changes including heart rate, blood pressure, and saturation varied with time, but there were no significant differences between the groups.

**Table 2:**
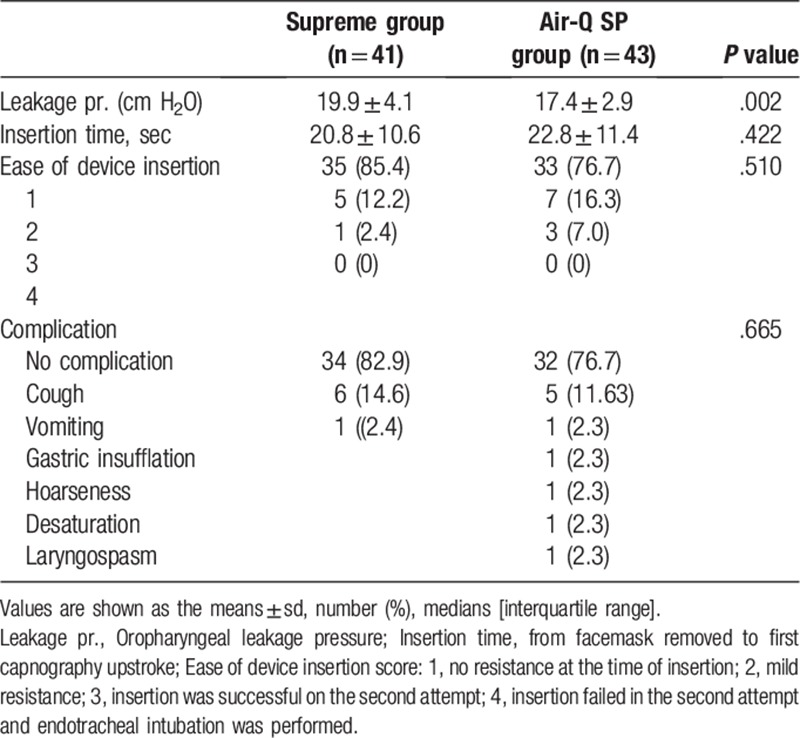
Comparison between the Supreme group and Air-Q SP group.

**Table 3:**
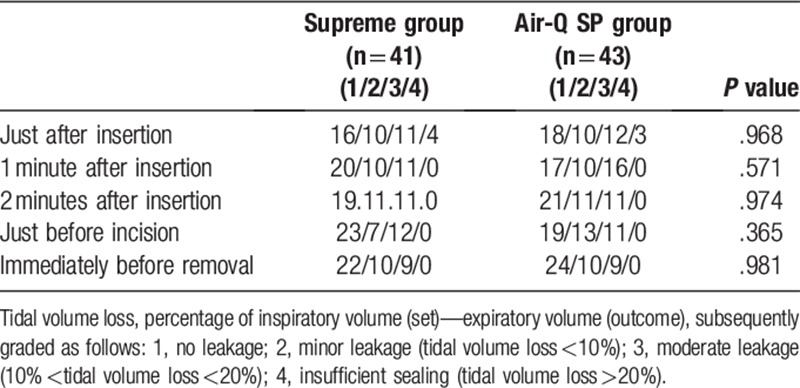
Tidal volume loss during mechanical ventilation.

Complications during the emergence (i.e., the process of recovery and awakening from anesthesia) are shown in Table 2. There were 7 complications in the Supreme group and 10 in the Air-Q SP group, but the difference was not significant (*P* = .665). In the Supreme group, there were 6 coughs and 1 vomiting episode, whereas in the Air-Q SP group there were 5 coughs and 1 vomiting episode. In the Supreme group, there was a suction hole that allowed for suction during vomiting. However, in the Air-Q SP group, because there was no suction hole, suction was often applied after device removal.

There was 1 case of gastric insufflation with abdominal distension in the Air-Q SP group. However, there was no tidal volume loss or desaturation in this patient, oropharyngeal leak pressure was 21 cm H_2_O, and airway pressure was maintained below 21 cm H_2_O. There was 1 case of hoarseness and 1 case of desaturation due to laryngospasm during emergence in the Air-Q SP group. Saturation was decreased to 70% and we had to remove the device early, and positive pressure ventilation was attempted and resolved without additional invasive airway procedures. In addition, there was 1 case in which saturation decreased by 60% because the patient had bitten the Air-Q SP and ventilation was limited. However, there were no further adverse events after early removal of the Air-Q SP.

## 4 Discussion

Our main finding suggests that the oropharyngeal leak pressure of the default setting of Supreme was non-inferior but rather superior. This means that despite not inflating the Supreme, it has better sealing function than that of Air-Q SP. However, there were no significant differences in tidal volume loss over time, ease of insertion, and insertion time between the 2 groups.

SGAs with noninflatable cuffs may reduce airway morbidity that is provoked by cuff hyperinflation; furthermore, they do not need to be measured for its intracuff pressure,^[11–13]^ so these are beneficial to children. Conventional SGAs with noninflatable cuff include Air-Q SP and I-gel.^[18,20,21]^ The Supreme originally consists of an inflatable cuff. However, in this study, we used the Supreme in the factory setting without any manipulation, such as deflation or additional air inflation when inserting the device. The cuff of Supreme was maintained slightly inflated state of the factory manufacturing settings.

The airway pressure at the point of air leakage around the device was expressed as oropharyngeal leak pressure, which is a measure of sealing function in the SGA.^[19]^ Sufficient sealing protects the larynx from oral secretions and enables safe and effective positive pressure ventilation.^[22]^ Brimacombe et al^[22]^ suggested that it is important to maintain the oropharyngeal leak pressure above 10 cm H_2_O. In our study, the oropharyngeal leak pressure remained above 10 cm H_2_O in all of the patients in the Supreme and Air-Q SP groups. In previous studies, Jagnnnathan et al^[18]^ showed no difference in oropharyngeal leak pressure between the Air-Q SP and laryngeal mask airway (LMA) Unique in children, and Galgon et al^[23]^ showed that the oropharyngeal leak pressure in the Air-Q SP and LMA Proseal were similar. However, in our study, the oropharyngeal leak pressure of the Supreme was significantly higher than that of the Air-Q SP group. We inserted the Supreme without deflation or additional air inflation (default setting), which differed from the manufacturer's instructions. The fact that oropharyngeal leak pressure of the Supreme without inflation (19.9 ± 4.1 cm H_2_O) was similar to a previous study reporting leak pressures of 20 cm H_2_O when with inflation,^[14]^ suggests that similar sealing function can be expected without air inflation.

The airway tube of the Air-Q SP is softer and more flexible than that of the Supreme and can affect resistance when inserted, but it can reduce insertion time since there is no deflation or additional air inflation process. Galgon et al^[23]^ reported that the Air-Q SP had a shorter insertion time than the LMA Proseal, but the ease of insertion was similar. Jagannathan et al^[18]^ showed that insertion success rates were similar, but that insertion was faster in the Air-Q SP group compared to the LMA Unique group. However, we found no significant difference in ease of insertion (insertion of device score) and insertion time between the 2 groups. This result may be due to the fact that we omitted the deflation and inflation process when inserting the Supreme, unlike the manufacturer's recommendations. In fact, there was more resistance, and a second attempt was required in the Air-Q SP group (3 cases) compared to the Supreme group (1 case); however, there were no significant differences. In a study by Kleine et al,^[23]^ the insertion time for the Supreme was 24 seconds; in our study, the shorter insertion time of 19 seconds appears to be due to the lack of any inflation process.

Overall complications were not significantly different between the 2 groups, but major complications such as laryngospasm, desaturation, and hoarseness were observed in the Air-Q SP group. This may be because there was no additional air inflation process when inserting the Supreme and we were able to prevent cuff hyperinflation. Since the airway tube of the Air-Q SP is softer than the Supreme, ventilation was difficult when the patient bites strongly during emergence. However, there were no additional adverse events after early removal of the Air-Q SP. Unlike the Supreme, the Air-Q SP did not have a drainage tube. Thus, there was gastric insufflation with abdominal distension in the Air-Q SP group, and suction was not possible with patient vomiting. In our study, there were many cases of cough in both groups, which may have been affected due to the use of lidocaine-based gels. The use of water-based lubricants may reduce these complications.

There were some limitations to our study. First, intracuff pressure was not measured after insertion of the Supreme. It is not important to measure the intracuff pressure because there was no deflation or additional air inflation when inserting the Supreme. However, it is important to confirm the changes in oropharyngeal leak pressure when additional air inflation is performed depending on intracuff pressure. Second, we studied healthy children of American Society of Anesthesiologists physical status 1 or 2; thus, the results of this study will not be applicable to children with impaired lung function or difficult airways. Finally, we did not confirm the fiberoscopic view after insertion of the Supreme and Air-Q SP, which should be performed in a future study.

In conclusion, the Supreme can be used in the default setting in pediatric patients accordingly in terms of tolerable leak pressure and the stability for mechanical ventilation compared with Air-Q SP.

## Author contributions

**Conceptualization:** Jagyung Hwang, Boohwi Hong, Yoon-Hee Kim, Yumin Jo, SooKyoung Youn, Chae Seong Lim.

**Data curation:** Jagyung Hwang, Boohwi Hong, Yumin Jo, SooKyoung Youn, Chae Seong Lim.

**Formal analysis:** Jagyung Hwang, Boohwi Hong, Chae Seong Lim.

**Investigation:** Yoon-Hee Kim, Chae Seong Lim.

**Methodology:** Jagyung Hwang, Boohwi Hong, Yumin Jo, SooKyoung Youn, Chae Seong Lim.

**Project administration:** Jagyung Hwang, Boohwi Hong, Yoon-Hee Kim, Yumin Jo, SooKyoung Youn, Chae Seong Lim.

**Resources:** Yumin Jo, Chae Seong Lim.

**Supervision:** Yoon-Hee Kim, Wonhyung Lee, Chae Seong Lim.

**Validation:** Boohwi Hong, Wonhyung Lee, Chae Seong Lim.

**Visualization:** Boohwi Hong, Chae Seong Lim.

**Writing - original draft:** Jagyung Hwang, Boohwi Hong, Yumin Jo, SooKyoung Youn, Chae Seong Lim.

**Writing - review & editing:** Jagyung Hwang, Boohwi Hong, Yoon-Hee Kim, Wonhyung Lee, Chae Seong Lim.

Chae Seong Lim orcid: 0000-0002-2356-8999.
